# Editorial: Nutraceuticals: New perspectives and approaches in human health and disease

**DOI:** 10.3389/fphar.2022.1003529

**Published:** 2022-09-02

**Authors:** Luigi Rosa, Maria Carmela Bonaccorsi di Patti, Vadim Vasilyev, Antimo Cutone

**Affiliations:** ^1^ Department of Public Health and Infectious Diseases, University of Rome La Sapienza, Rome, Italy; ^2^ Department of Biochemical Sciences, University of Rome La Sapienza, Rome, Italy; ^3^ Institute of Experimental Medicine, Saint Petersburg, Russia; ^4^ Department of Biosciences and Territory, University of Molise, Pesche, Italy

**Keywords:** nutraceuticals, dietary supplements, herbal products, nutrients, milk proteins

A nutraceutical is defined as a “food (or a part of food) that provides medical or health benefits, including the prevention and/or treatment of a disease” (Das et al., 2012). The term is applied to all components, or active ingredients, derived from foods of animal, vegetable or microbial origin, such as milk proteins, herbal products, phytochemicals and probiotics, that, other than nutrition, exhibit beneficial effects for human health. Recently, the interest in nutraceuticals is growing as most of them possess multiple therapeutic properties, including anti-microbial, anti-oxidant, anti-inflammatory and anti-cancer ones. Importantly, these products are usually recognized as “safe” and less likely to exert side effects.

Most nutraceutical intake occurs orally, and bioavailability varies greatly depending on several factors, such as physico-chemical properties, pH resistance and absorption rate. One of the main challenges for nutraceutical research relies on the effort to maximize both source quality and body delivery. Indeed, in the last decades, the optimization of nutraceutical formulations has greatly implemented the manufacturing and marketing in both pharmaceutical and food industry. On the other hand, food products for nutritional purposes are not regulated like licensed medicines and scientific research is often misinterpreted or overstretched for commercial interests. Therefore, it is imperative to re-evaluate basic research, concerning structure-function relationship, molecular interactions, activation/inhibition of cellular signaling, to reveal the actual (in) efficacy of such products on human health and disease.

A total of 11 manuscripts were published in this Research Topic including six reviews and five original research articles.

Two reviews have highlighted the role of colostrum and its components in human health. In the manuscript by Kaplan et al., the Authors critically analyze the industrial processes that can influence the composition and the nutritional values of bovine colostrum, and introduce new advanced technologies in the field aimed at preserving the quality of the end-products (Kaplan et al.). In the review by Ramirez-Rico et al., lactoferrin (Lf), an iron-binding glycoprotein primarily found in milk, is presented as a promising compound able to regulate the inflammatory response and maintain gut homeostasis, thus counteracting and potentially preventing colorectal cancer as well reducing, as an adjuvant, side effects of chemotherapy (Ramirez-Rico et al.).

Pharmaceutical interest in the human intestinal microbiota has increased considerably, in the light of studies linking the human intestinal microbial ecology to an increasing number of non-communicable diseases. Many efforts in modulating gut microbiota have been made by using probiotics, prebiotics and, recently, postbiotics. The review by Spisni et al. summarizes all *in vitro*, *in vivo* and clinical studies in such a field, demonstrating the efficacy of these preparations in intestinal microbial homeostasis, by selectively acting on pathobionts, without altering the portion of health-associated commensals (Spisni et al.). In the study by Brunelli et al., human cell line models were used to assess the potential capacity of AminoAlta™ probiotic formulation (AApf) to protect from the physiological damages that an intense physical activity may cause. The obtained results revealed that the bacteria present in the AApf have the ability to reduce trans-epithelial permeability and decrease NF-κB signaling in Caco-2 cells under inflammatory stimulation as well as to trigger in macrophagic THP-1 cells the expression of cytokines, such as IL-1β, IL-6, and TNF-α, which typically intervene in counteracting bacterial and viral infections (Brunelli et al.). Some genetically modified bacteria, including probiotics, represent attractive vehicles for oral or nasal mucosal delivery of therapeutic molecules. It is the case of Suvorov et al. who present a study demonstrating the construction of the novel SARS-CoV-2 vaccine candidate employing the gene fragment of S1 SARS-CoV-2 gene. Expression data allowed to consider such genetically modified probiotic strain as an interesting candidate for vaccine against SARS-CoV-2 (Suvorov et al.). Of note, metabolites produced by the intestinal microbiota have shown to play a role in human health. In the review by Amiri et al., a potentially therapeutic role for butyrate, a bacterial secondary metabolite, in cardiovascular diseases and the mechanisms and pathways involved in the cardio-protective effects were deeply analyzed. In summary, the Authors indicate that although butyrate exhibits a wide variety of biological activities towards different cell and biochemical pathways, such as energy homeostasis, glucose and lipid metabolism, inflammation, oxidative stress and neural signaling, it remains unclear whether these findings are clinically relevant (Amiri et al.). Also Vitamin K2-7, a form of vitamin K produced by intestinal bacteria, has been demonstrated to exert health-beneficial effects in different pathological conditions, such as osteoporosis, cardiovascular disease, inflammation, cancer, Alzheimer’s disease, diabetes and peripheral neuropathy, as reviewed by Jadhav et al.

Extra virgin olive oil (EVOO) from *Olea europaea*, a cornerstone in the Mediterranean diet, is well known for its nutritional and health properties, especially for prevention of cardiovascular diseases and metabolic disorders. In the paper by Di Pietro et al., the antimicrobial activity of different green extra virgin olive oil-based formulations in natural deep eutectic solvents (NaDESs) was demonstrated. Specifically, the EVOO extract showed the highest antibacterial activity against several clinical strains of *Staphylococcus aureus*, whereas oleacein in was the most effective toward various clinical strains of *Escherichia coli*, *Pseudomonas aeruginosa*, and *Klebsiella pneumoniae* (Di Pietro et al.). Another biologically active nutraceutical from plants is resveratrol. The study by Modzelewska et al. provides the first observation that resveratrol exerts relaxant effects in human gastric muscle strips by activating calcium-activated potassium (BK_Ca_) channels independently of nitric oxide signaling pathways, opening new frontiers in resveratrol applications as in the treatment of gastrointestinal dyspepsia and other gastric hypermotility disorders (Modzelewska et al.).

The effect of natural products on hepatic fibrosis has been reviewed by Shan et al., highlighting the potential combination of different drugs, both of natural and recombinant origin, which have shown advantages of improving efficacy and reducing toxicity in clinical studies. Within this frame, the study by Jiang et al. has demonstrated the efficacy of new antioxidant compounds in ameliorating liver dysfunction via reducing iron in a murine model of thioacetamide-induced acute liver injury. In particular, along with the reduction of hepatic iron accumulation, the decrease of serum ALT, AST and LDH levels as well as the physiological rebalance of iron-proteins expression were observed (Jiang et al.).

The Research Topic “Nutraceuticals: New Perspectives and Approaches in Human Health and Disease” was effective in putting together new and promising studies on the subject, highlighting the potential application of natural products in current and future pharmacological research, as actual alternative or complement to standard pharmacological therapy.

## References

[B1] Das L. Bhaumik E. Raychaudhuri U. Chakraborty R. (2012). Role of nutraceuticals in human health. J. Food Sci. Technol. 49, 173–183. 10.1007/s13197-011-0269-4 PubMed Abstract | 10.1007/s13197-011-0269-4 | Google Scholar 23572839 PMC3550857

